# CTXA Hip—An Extension of Classical DXA Measurements Using Quantitative CT

**DOI:** 10.1371/journal.pone.0091904

**Published:** 2014-03-17

**Authors:** Christopher E. Cann, Judith E. Adams, J. Keenan Brown, Alan D. Brett

**Affiliations:** 1 Mindways Software, Inc., Austin, Texas, United States of America; 2 Department of Radiology, University of California San Francisco, San Francisco, California, United States of America; 3 Department of Clinical Radiology, University of Manchester, Manchester, United Kingdom; University of Rochester, United States of America

## Abstract

Bone mineral density (BMD) estimates for the proximal femur using Dual Energy X-ray Absorptiometry (DXA) are currently considered the standard for making a diagnosis of osteoporosis in an individual patient using BMD alone. We have compared BMD results from a commercial Quantitative CT (QCT) BMD analysis system, “CTXA Hip”, which provides clinical data for the proximal femur, to results from DXA. We have also used CTXA Hip to determine cortical and trabecular contributions to total BMD. Sixty-nine patients were scanned using 3D QCT and DXA. CTXA Hip BMD measurements for Total Hip and Femoral Neck were compared to DXA results. Twenty-two women were scanned at 0,1,2 years and CTXA Hip and DXA results analyzed for long-term reproducibility. Long-term reproducibility calculated as root-mean-square averages of SDs *in vivo* was 0.012 g/cm^2^ (CV = 1.8%) for CTXA Total Hip and 0.011 g/cm^2^ (CV = 2.0%) for CTXA Femoral Neck compared to 0.014 g/cm^2^ (CV = 2.0%) and 0.016 g/cm^2^ (CV = 2.7%), respectively, for DXA. The correlation of Total Hip BMD CTXA vs. DXA was R = 0.97 and for Femoral Neck was R = 0.95 (SEE 0.044 g/cm^2^ in both cases). Cortical bone comprised 62±5% (mean ± SD) of total hipbone mass in osteoporotic women. CTXA Hip provides substantially the same clinical information as conventional DXA and in addition provides estimates of BMD in separate cortical and trabecular bone compartments, which may be useful in evaluation of bone strength.

## Introduction

Osteoporosis is a major public health concern. It is estimated that up to 50% of women and 20% of men in the US are at risk for developing an osteoporosis-related fracture during their lifetime [1]. Bone mineral density (BMD) estimates may be used to test for osteoporotic fracture risk and to make decisions about the initiation of pharmacologic therapy. According to the official positions of the International Society for Clinical Densitometry (ISCD) [2] and the National Osteoporosis Foundation (NOF) [3], central DXA of the lumbar spine and proximal femur is the preferred method for bone mineral density (BMD) testing. Osteoporosis is diagnosed by central DXA in postmenopausal women and in men aged 50 years and older if the T-score of the lumbar spine or hip is −2.5 or less. Low bone mass or osteopenia is classified as a T-score of −1.0 or less [2].

Despite the fracture risk statistics, osteoporosis testing with dual-energy X-ray absorptiometry (DXA) remains underused [4]. However, BMD can also be assessed with other radiologic imaging tools, such as quantitative computed tomography (QCT) that may be available when access to DXA is restricted.

Historically, in spite of QCT having a number of advantages over DXA [5], the use of QCT was limited by a number of factors including the cost and availability of early CT scanners, and the time required for analysis using the first QCT systems. In addition, the original implementations of 2D QCT BMD measurement at the spine tended to show lower precision due to the operator dependence of 2D scan protocols based on tilting a CT gantry, and QCT is associated with a relatively high x-ray dose compared to DXA. However, modern MDCT machines are both fast and widely available, and have allowed the introduction of 3D QCT volumetric scan protocols, reducing operator dependence and enabling CV precision errors of 0.8% [6]. Contemporary low-dose CT techniques directed at focused regions of interest and utilizing Automatic Exposure Control (AEC) can minimize radiation exposure [7].

While DXA of the spine computes an “areal BMD” (aBMD) measurement by the projection of integral (cortical and trabecular) vertebral bone onto a 2D plane, QCT provides a volumetric BMD measure of the trabecular vertebral bone in isolation. This can have an advantage of superior sensitivity due to the higher metabolic rate of turnover of trabecular bone [5]; and can also avoid the confounding effects of joint-space narrowing, osteophytes, aortic calcification and other extra-osseous calcification that can artificially raise a DXA spine BMD measurement [8]–[11]. However, the measurement of isolated trabecular bone means that QCT vertebral T-scores are somewhat lower than DXA T-scores for the same age [12] and application of the established WHO classification of osteoporosis by DXA T-score is not appropriate. In order to facilitate the interpretation of QCT spine results, the American College of Radiology has in 2008 published guidelines for the performance of QCT [13]; based on these guidelines, volumetric trabecular BMD values from 120 to 80 mg/cm^3^ are defined as osteopenic and BMD values below 80 mg/cm^3^ as osteoporotic.

In addition to the spine, the hip is an important fracture risk site and therefore an important site for axial BMD measurement [14] and areal BMD measurement at the femoral neck is one of the clinical risk factors included in the WHO FRAX 10-year fracture risk calculation tool [15]. While 3D QCT systems for measurement at the hip have been proposed and developed for research [16], the volumetric BMD measurements from these tools lack the normal comparative data that exists for lumbar spine QCT.

CTXA Hip uses 3D QCT volume data sets to generate bone projection images that visually look like those generated by DXA. CTXA Hip exploits the anatomical detail in the 3D QCT data set to segment bone from surrounding tissues rather than relying on the dual-energy imaging method of DXA. While CTXA Hip and DXA use somewhat different technologies to generate bone projection images of the proximal femur, the projection images from both devices convey the same basic information—total bone mass per projected bone area. This leads to the hypothesis that CTXA Hip BMD estimates provide the same clinical utility as that afforded by DXA. Importantly, the areal BMD measurements and T-scores derived from CTXA Hip may be used with the WHO diagnostic classifications.

This kind of approach has been shown to have excellent correlation with DXA for areal BMD measurement for the Total Hip region [17]. In a previous study using CTXA Hip, Khoo et al compared it with DXA-derived areal bone mineral density and T-scores [18]. However, that study looked at a cohort of elderly patients aged 82.8+/−2.5 years (mean +/− SD) and did not utilize CTXA to derive separate measurement from cortical and trabecular compartments; CTXA Hip may provide more information than DXA from a study due to the greater anatomical detail accessible from the 3D QCT volume data set relative to the information present in the planar projection images intrinsic to DXA.

In this study, we compare CTXA Hip and DXA areal BMD measures at both the femoral neck and total hip regions in a cohort of women between the ages of 20–80 years. We also present cortical and trabecular BMD estimates from standard (DXA) hip ROIs derived using CTXA Hip as additional information available from this method.

## Materials and Methods

QCT studies were performed using the QCT Pro calibration phantom and software system with the CTXA Hip analysis module (Mindways Software, Inc., Austin, TX). The Mindways liquid calibration phantom used in this study was based on the original QCT calibration system developed at University of California, San Francisco (UCSF) [19], and mineral density results are reported in terms of equivalent calibrated aqueous potassium phosphate density. The QCT Pro QA phantom included a reference tube that contains aqueous K_2_HPO_4_ with a concentration of 200.0±0.4 mg/cm^3^, and was used within the software both as a cross-calibration reference and for monitoring scanner performance.

**Figure 1:**
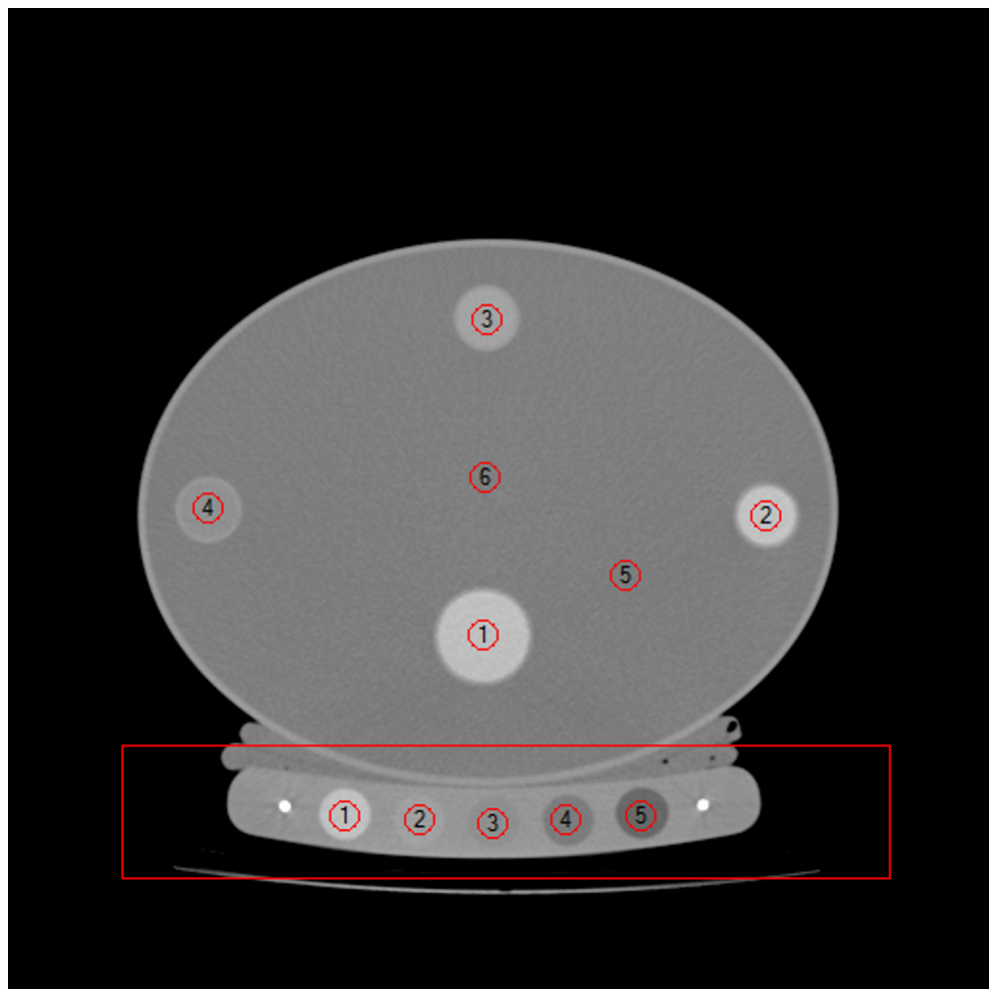
QA study axial image. Completed ROI placement in axial CT slice of quality assurance and CT calibration reference phantoms positioned for QA studies.

Quality assurance (QA) scans were performed once a month according to manufacturer's protocols. In brief, the QA phantom was placed above the calibration phantom and 8–10 axial slices were scanned using the same study protocol used for BMD studies as described below, see Figure 1.

### Subjects

#### Precision Study

A total of 22 subjects enrolled as placebo controls in an osteoporosis treatment study at the University of California, San Francisco (UCSF) comprised the precision study group. These “placebo” subjects were all postmenopausal osteoporotic women receiving hormone replacement therapy, calcium and vitamin D, and ranged in age from 55 to 72 years. All women were enrolled in that study by being identified as “osteoporotic” based on a spine or hip BMD T-score by DXA less than −2.5. Hip BMD was measured at yearly intervals for each subject using DXA and QCT. Ten of the 22 women received three hip BMD scans over a two year interval while 12 of the women received four hip BMD scans over a three year interval.

#### Interobserver Variability

The interobserver variability for BMD estimates using CTXA was estimated by comparing results obtained independently on the same in vivo data set by two trained operators. Data from a single clinical trial site were used for this analysis. Twenty eight studies were analyzed, and the results for estimated BMD of the femoral neck and total hip regions of interest were compared.

### CTXA-DXA Comparison

A total of 69 patients having DXA exams for osteoporosis diagnostic testing were recruited from two clinical centers, the University of Manchester, UK, and Schenectady Radiology/Ellis Hospital, Schenectady, NY, for comparison of CTXA Hip and DXA results. Thirty patients were recruited at one center and 39 patients at the other. Seven patients were men and 62 were women. Ages ranged from 20–80 years, but the patients were predominantly postmenopausal women.

### Ethics

All subjects involved in this study gave written informed consent, and all research protocols were approved by the Institutional Review Boards at the respective institutions: the Institutional Review Board of Ellis Hospital, Schenectady, NY, USA; the Committee on Human Research (CHR), the UCSF Institutional Review Board holding Department of Health and Human Services Multiple Project Assurance #M-1169, University of California, San Francisco, USA; and the Ethics Committee of the University of Manchester, Manchester, UK.

### Scanning Protocols

#### CTXA Examinations

CT image data analyzed for these studies using CTXA Hip were acquired using different CT scanner models at the three institutions. At UCSF, a GE9800 (GE Medical Systems, Waukesha, WI) was used, at the University of Manchester a Philips SR4000 (Philips Medical Systems, Best, Netherlands) was used, and at Schenectady Radiology a GE ProSpeed (GE Medical Systems, Waukesha, WI) was used. All CT scanners were maintained as specified by the manufacturers. In addition, all CT systems were further calibrated for QCT using the QCT Pro QA procedures.

Subjects were positioned supine on the CT scanner table, lying on top of a K_2_HPO_4_ CT calibration phantom and bolus bags so that the calibration phantom extended from the lumbar vertebrae to mid-thigh, to cover the pelvis and proximal femur region. Positioning was used so that the pelvis was as straight as possible and the knees were flat on the scanner table. Subjects were asked to put their feet together and remain still, but the feet were not restrained by a positioner. An anterior-posterior computed radiograph was obtained by the scanner from the iliac crest to mid-thigh, and the top of the femoral head to approximately 1 cm below the inferior extent of the lesser trochanter was defined graphically to define the scanning region. A contiguous series of scans was obtained, 3 mm thick every 3 mm, with a 40 cm display field-of-view (0.781 mm pixel size), and a standard abdomen reconstruction algorithm. Typically 40 images were obtained, with the time to acquire this image set approximately 3.5 minutes on the GE9800 and 1 minute on the Philips and ProSpeed scanners. Scanning parameters varied slightly depending on the capabilities of the CT scanner used and patient size, and were 80 kVp, 240 mAs for the GE9800, and 120 kVp, 100–200 mAs for the Philips and ProSpeed scanners. All subject and QA data were sent to Mindways where analysis was centralized.

#### DXA Examinations

DXA image data were acquired and analyzed at each site according to standard procedures used at those sites, including daily calibrations for quality control. A single individual at each site was responsible for all DXA analyses. At UCSF, DXA data were acquired using a Hologic QDR1000 (Hologic, Inc., Bedford, MA) scanner, while at the other two sites Hologic QDR4500 scanners were used. The DXA systems were calibrated and maintained in accordance with the manufacturer's specifications. The DXA data were acquired and analyzed according to the manufacturer's instructions.

### Data Analysis

QA phantom data for each CT scanner were analyzed using the QCT Pro QA analysis module. Each QA study of 8–10 QA images acquired using the same technique as for subject scans was analyzed to determine CT scanner performance characteristics, and any deviations from expected performance were identified by the software. Any degradation of scanner performance identified by the QA software was resolved before subsequent subject data were analyzed. Subject results were referenced to the appropriate CT scanner QA results.

The QA phantom data is also used by the CTXA Hip software to detect and characterize differences in CT value response and make a correction to the CT calibration slope to compensate for “beam hardening” effects.

**Figure 2:**
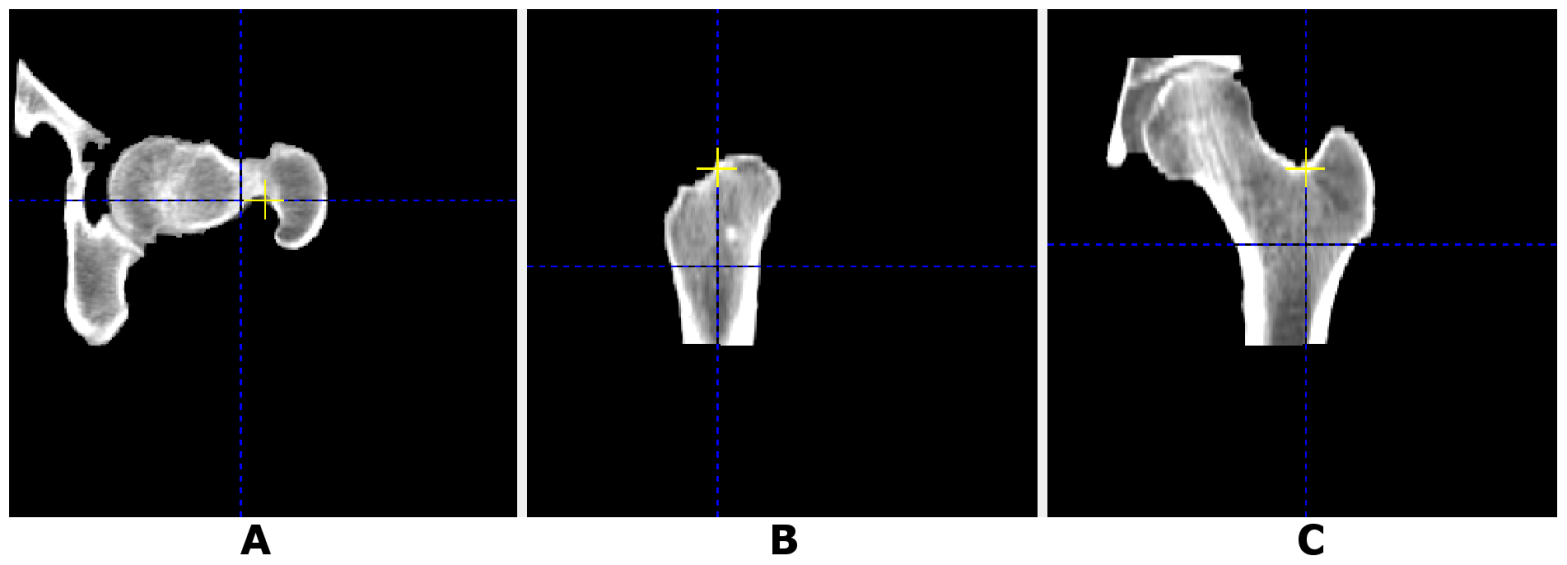
Proximal femur positioning. A) axial, B) sagittal and C) coronal images of segmented bone in proximal femur, rotated into a standard projection.

CT image data were analyzed in a standardized fashion with the CTXA Hip software, using the left proximal femur unless pathology prevented this. A square box region of interest was centered over the femoral neck as identified on the axial images, and a volumetric region of interest containing the proximal femur was extracted from the CT image data set for analysis. Segmentation of bone from surrounding non-bone tissue was performed using an adaptive algorithm controlled by three parameters. The first parameter defined a threshold below which a pixel could not be classified as bone (default: −250 mg/cm3). The second parameter defined the size of the neighborhood of pixels considered when adaptively modifying the local threshold. A default range corresponds to a neighborhood with a width of approximately five pixels. The third parameter was the initial threshold used to seed the adaptive algorithm, with pixels exceeding the initial threshold tentatively classified as “bone” and the remainder as “not bone” (default: 120 mg/cm3). The result of the segmentation process is a set of voxels identified as “bone” all contained within the outer cortex of the proximal femur. There was no further separation of voxels within this outer envelope as “marrow” vs. “bone” voxels. This 3D data set of bone voxels was then rotated such that the femoral shaft was vertical in the coronal and sagittal planes and the femoral neck was horizontal in the axial plane (Figure 2).

**Figure 3:**
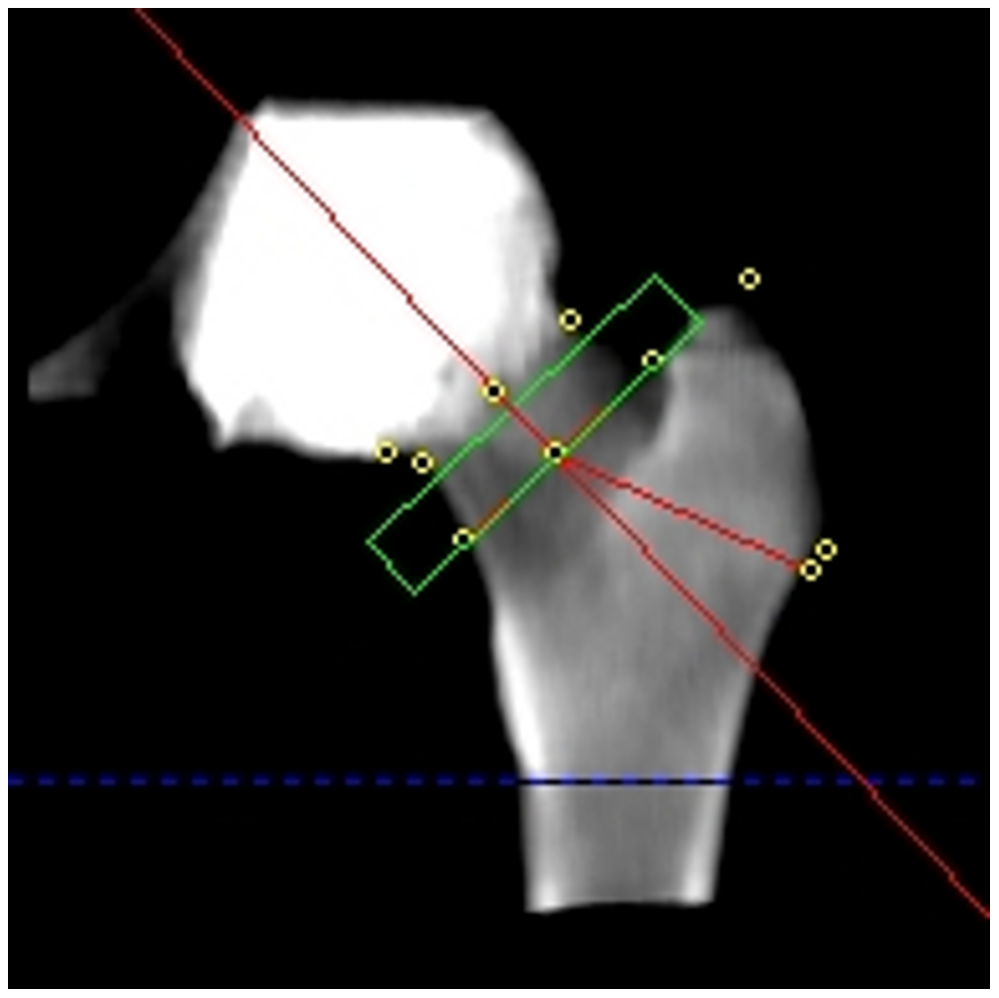
CTXA proximal femur ROI positions. CTXA projected image with standard regions of interest used for BMD calculations (femoral neck, trochanter, intertrochanter, and Total Hip as sum of these three regions). Position of femoral neck box and intertrochanter limit line at base of lesser trochanter, and rotation of femoral neck axis, are adjustable by user. Ward's Triangle ROI is displayed but not used in comparisons.

The CTXA Hip software generates a 2-dimensional image similar to a DXA image from the rotated 3D data set by summing all the bone voxels along lines perpendicular to the coronal plane. Each pixel of the resulting image represented the mass of mineral summed along that line, and was further characterized by a known pixel area, and a total volume of bone along the line. Regions of interest representing the common ROIs used for DXA analysis (Total Hip, Femoral Neck, Trochanter, Intertrochanter) were identified automatically on the projected image by the software (Figure 3). The lower extent of the Intertrochanter ROI was set at the lower junction of the lesser trochanter and the femoral shaft. The angle of the femoral neck axis, and the position and size of the femoral neck box ROI, were adjusted by the operator as required.

Results for areal BMD (g/cm^2^), volumetric BMD (mg/cm^3^), mass (g), area (cm^2^) and volume (cm^3^) for each of the ROIs were stored in the QCT Pro database and exported as text files for analysis.

In addition to the primary data analysis of total bone in the projected ROIs, the software also allows classification of bone voxels as “cortical” or “trabecular” based on a simple user-defined threshold. A threshold of 350 mg/cm^3^ was used to separate “cortical” from “trabecular” bone in these ROIs. This value was chosen empirically as that which compartmentalized the major compressive and tensile trabecular bone groupings as “trabecular” bone. The choice of this threshold also identified higher density endocortical bone as “cortical” even though it was not part of the haversian cortex. The same measurements of area BMD, volumetric BMD, mass, area, and volume were given for each of these compartments. Compartmental analysis of the ROIs was performed for the group of 22 women recruited for the precision study as a homogeneous population of postmenopausal women identified as osteoporotic by DXA.

### Statistical Analysis

The precision of area BMD (expressed in g/cm^2^) measurements derived with the CTXA Hip was estimated by examining the dispersion of patient measurements acquired at approximately yearly intervals. The mean and standard deviation for the 3 or 4 measurements for each patient were calculated without regard for the rate of bone change. Long-term precision was then estimated by calculating the root-mean-square average of the set of standard deviation estimates for the group of patients [20]. This method provides a conservative estimate of precision, and is justified because the women in this group on average were not losing bone (CTXA Hip Total Hip BMD mean and SEM for baseline, year 1 and year 2, 0.647±0.017, 0.644±0.018, 0.648±0.019 g/cm^2^, p>0.05 for all comparisons by two tailed t-test).

For interobserver variability, significance of difference of means was tested using a two-tailed t-test.

The accuracy of the CTXA Hip BMD estimates relative to Hologic QDR DXA BMD estimates for the Total Hip and Femoral Neck ROIs was characterized by comparing CTXA Hip and DXA results from the same subject from the two sites using Hologic QDR4500 scanners. Results were first compared by individual site. Similarity of the distributions suggested pooling the results. Pooling of these results was objectively justified based on a two-sample t-test, assuming unequal variances, of the means of the bias distribution for each site. That is, the means of the distribution of patient-specific CTXA Hip minus DXA BMD results determined from paired subject measurements at each of the sites were compared by this method. No inconsistencies in the sample mean comparisons were found either for the total hip or femoral neck data at the 95% confidence level (p<0.05).

To ascertain the validity of t-tests, an Anderson-Darling test was used to detect significant deviations from normality in measurement distributions.

## Results

The results of the long term in vivo precision studies are given in Table 1 (Table S1: “LongTerm In Vivo Precision CTXA vs DXA - Data”), both for precision in terms of the BMD value (g/cm^2^) and as the coefficient of variation (CV,%), based on the distribution of the individual patient values. The mean BMD values by CTXA Hip and DXA are also given. There were no significant differences in precision between the CTXA Hip and the DXA results obtained in this study.

**Table 1:** Summary of Long-Term *In Vivo* Precision, CTXA vs. DXA, in Osteoporotic Subjects.

	Total Hip		Femoral Neck	
	CTXA	DXA	CTXA	DXA
Areal Density (g/cm^2^)	0.645	0.700	0.551	0.598
Precision (g/cm^2^)	0.012	0.014	0.011	0.016
CV (%)	1.8	2.0	2.0	2.7

Interobserver variability results for CTXA Hip are given in Table 2 (Table S2: “Interobserver Comparison of CTXA BMD Estimates - Data”). The difference in total hip mean BMD estimates was not significant between the two observers at the 95% confidence level (p = 0.055), while the femoral neck difference was significant between the two observers at the 95% confidence level (p = 0.026). Even though statistically significant, the 1% difference between observers is similar to the 0.9–2.6% obtained using DXA (6,7).

**Table 2:** Interobserver Comparison of CTXA BMD Estimates (g/cm^2^).

	Observer 1	Observer 2	Observer 2 - Observer 1	Observer 2 - Observer 1 (%)
Mean Total Hip BMD	0.668	0.675	0.007	1.0
SD Total Hip BMD	0.142	0.146	0.004	
Mean Femoral Neck BMD	0.585	0.578	−0.007	1.2
SD Femoral Neck BMD	0.121	0.118	−0.003	

No substantial evidence was found for rejecting the hypothesis that the data measurements are reasonably described by a normal sampling process using the Anderson-Darling test to detect significant deviations from normality in measurement distributions.

**Figure 4:**
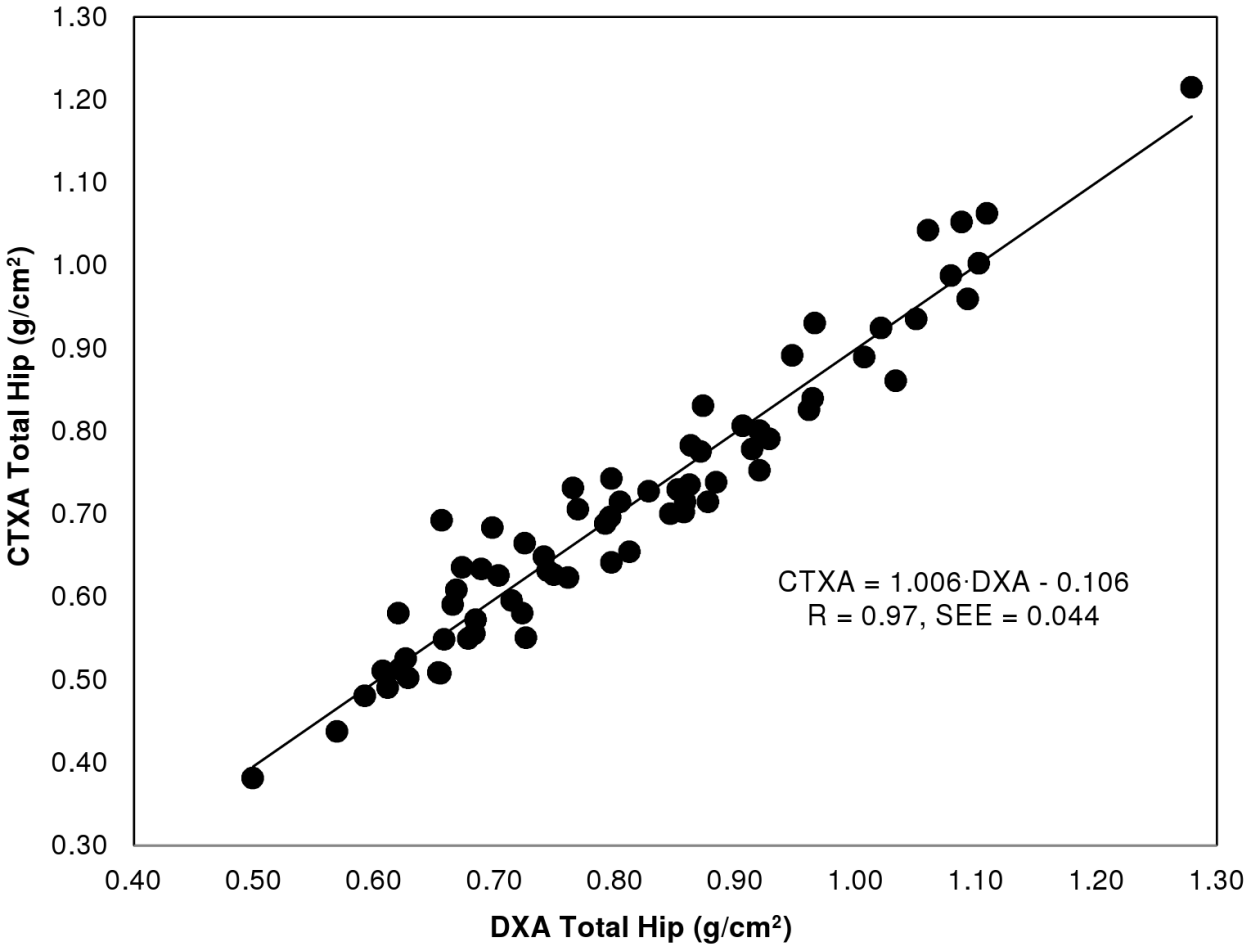
Correlation of area BMD for CTXA and DXA for total hip region of interest.

**Figure 5:**
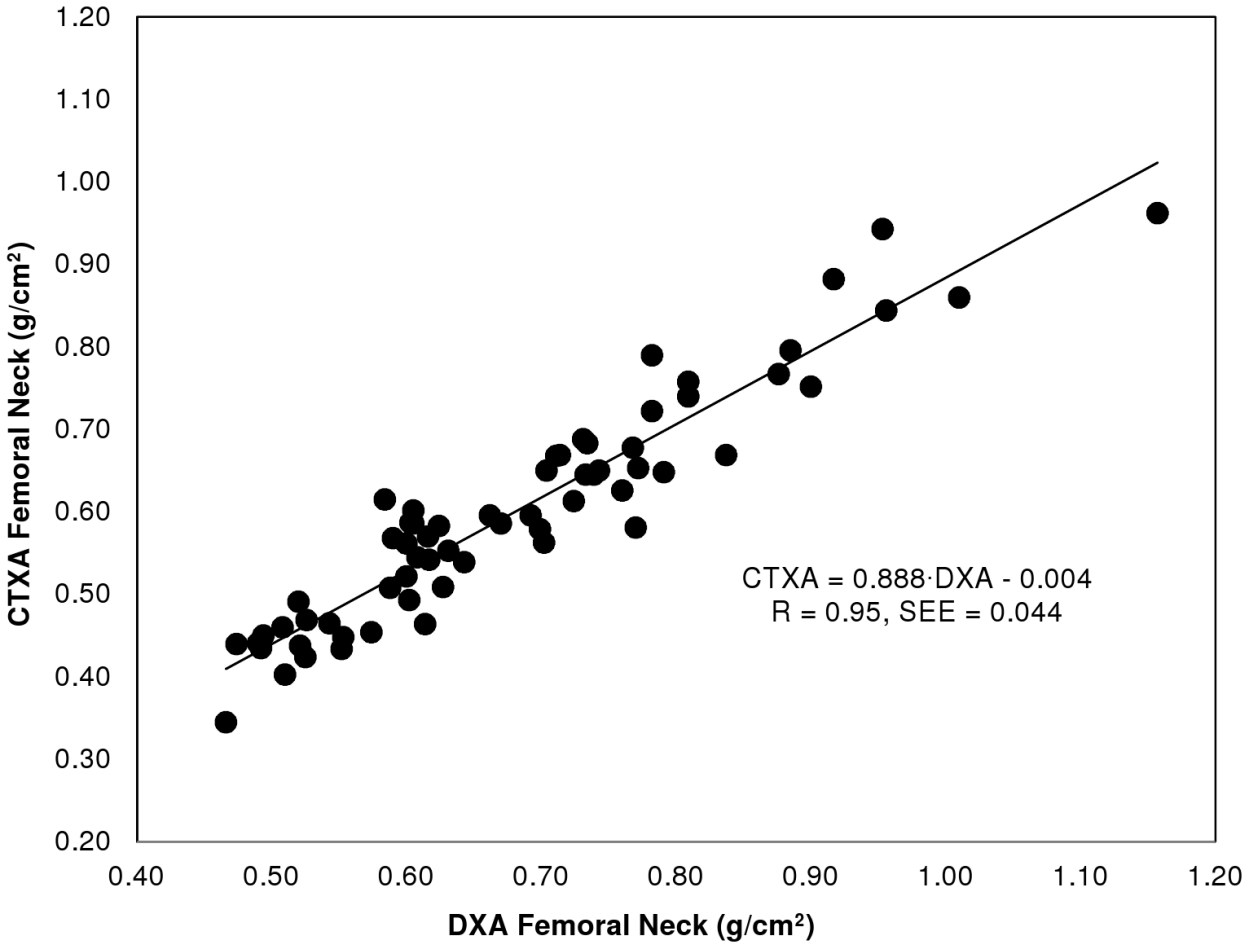
Correlation of area BMD for CTXA and DXA for femoral neck region of interest.

The correlation between BMD estimates made with CTXA Hip and DXA for total hip and femoral neck regions of interest are given in Table 3 (Table S3: “Correlation of CTXA Hip and DXA BMD results - Data”), for the two clinical sites independently and for pooled results. Figures 4 and 5 show the correlations for total hip and femoral neck graphically. Correlation coefficients of 0.92−0.97 were obtained for femoral neck and total hip ROIs, with Standard Error of the Estimates (SEE) of 0.043−0.047 g/cm^2^.

**Table 3:** Correlation of CTXA Hip and DXA BMD Results (Site 1, Manchester; Site 2, Schenectady).

Total Hip						
	CTXA BMD	DXA BMD	Slope	Intercept	R	SEE
Site 1 (N = 30)	0.657±0.143	0.766±0.138	0.988	−0.100	0.95	0.046
Site 2 (N = 39)	0.755±0.175	0.851±0.170	0.999	−0.094	0.97	0.043
Site 1+2 (N = 69)	0.712±0.168	0.814±0.161	1.006	−0.106	0.97	0.044

Femoral Neck						
	CTXA BMD	DXA BMD	Slope	Intercept	R	SEE
Site 1 (N = 30)	0.564±0.117	0.645±0.123	0.876	−0.001	0.92	0.047
Site 2 (N = 34)	0.638±0.150	0.713±0.156	0.883	0.004	0.96	0.043
Site 1+2 (N = 64)	0.606±0.141	0.681±0.145	0.888	−0.004	0.95	0.044

The segmentation of total bone in the hip into compartments representing “cortical” and “trabecular” bone in DXA-like ROIs differentiates the measurement results of CTXA Hip analysis from DXA. For the study population, the total hip region of interest was found to contain 62.3%±4.8% (mean ± SD) “cortical” bone with the remaining 37.7%±4.8% bone belonging to the “trabecular” bone compartment defined by CTXA Hip. For the femoral neck the proportions were 57.7%±9.5% and 42.3%±9.5%, respectively.

## Discussion

The focus on measurements of bone density at the proximal femur as a standard reference [14] has meant DXA has become the gold standard technology used to make these measurements. Theoretically QCT can produce a BMD estimate at the hip in two dimensions that has characteristics similar to DXA, and should give a BMD estimate that is highly correlated with a DXA result. The results of our study show that 2D projected BMD results obtained using the CTXA Hip software correlated highly with results obtained from a common DXA device (QDR4500), and these results are similar to those from other studies. Khoo et al, [18] showed correlation between DXA and CTXA of R = 0.89–0.93 in an older cohort, with a RMS errors of between 0.04–0.06 g/cm^2^. In a comparison of a their own QCT aBMD projection algorithm for Total Hip region, Keyak et al [17] showed a correlation of R = 0.935 with an SEE of 0.046 g/cm^2^.

The results obtained in our study are also similar to the relationship seen between DXA systems from Hologic and Lunar, where a correlation of R = 0.92 and SEE of 0.051 for the Femoral Neck ROI over the same BMD range in a similar population has been reported [21], [22].

Long term in vivo precision is an important parameter for clinical practice, and we obtained results for CTXA Hip essentially identical to those from a Hologic QDR1000 system for osteoporotic patients studied under controlled conditions over a 2–3 year period. Our long-term precision in osteoporotic subjects is similar to short-term precision of 2.1–2.9% obtained by other researchers using DXA in similar populations [23], [24], indicating that when properly performed QCT methods are just as precise as DXA. Our precision results are consistent with the observations of Khoo et al [18] where CTXA short-term precision estimates that were either non-inferior to or superior to DXA were reported. However, a limitation of the present study is that the results from only two observers where available for the reproducibility analysis.

We observed statistically significant differences in the slope and/or intercept of BMD results from CTXA Hip compared to Hologic DXA. In particular, CTXA Hip BMD estimates for the Total Hip ROI were found to be approximately 0.11 g/cm^2^ lower than QDR 4500 results for the same population. This bias is well modeled by an additive (negative) bias term as shown in Figure 4 where the observed slope in the linear regression analysis was found to be not significantly different from unity. CTXA Hip BMD estimates for the Femoral Neck were also found to be less than the corresponding QDR 4500 results with an average bias of about 0.06 g/cm^2^. In this case, however, linear regression analysis indicated the bias was better explained by a model slope significantly different than one with an additive offset not significantly different from zero. As can be seen in Table 1, we also observed statistically significant bias in CTXA Hip and QDR 1000 BMD estimates, with an average bias of about 0.05 g/cm2 at both measurement sites reported here for the osteoporotic patient population comprising our precision study group.

Biases in hip BMD estimates between bone densitometers from various manufacturers have been reported in numerous studies. Biases of the same magnitude we report here have been observed in comparison studies of DXA devices from Hologic, Lunar and Norland [21], [22]. As noted by those authors, these differences may be due to technical differences in the way data are acquired and analyzed.

One cause of the differences between QCT (CTXA) aBMD and DXA-derived aBMD relates to the calibration phantoms used [25]. The Hologic DXA scanner uses solid calcium hydroxyapatite (CaHAP) standard for its calibration. QCT uses liquid potassium phosphate (K_2_HPO_4_) as a mineral standard. Although the mean CaHAP and K_2_HPO_4_ calibration standards are similar in the trabecular aBMD region, there are slight differences in the calibration slopes, with a propensity for K_2_HPO_4_ equivalent densities to be slightly lower than corresponding CaHAP equivalent densities. The difference is more pronounced when working at higher densities. Therefore, it is not surprising that QCT-derived mineral mass (BMC) at all corresponding sites of the proximal femur was significantly lower compared to DXA values (Table 1). In addition, the methods used for separating “soft tissue” components of the projection are also very different between QCT and DXA. DXA uses dual energy methods for this and in particular estimates the fat/lean ratio for tissue superimposed with the DXA bone signal. Essentially the soft-tissue component is subtracted in DXA. In CTXA, however, volumetric CT images are segmented into “bone” and “not bone” (mostly soft tissue, air and calibration phantom pixels) pixels using geometric, anatomical and physiological considerations. The “non-bone” pixels are completely removed from the image data prior to generating bone projections and so contribute essentially no signal (and no residual noise) component to the bone projection images and without dependencies on details of the nature and distribution of the surrounding tissue.

Next, DXA standardizes measurement positioning by controlling foot positioning during scanning. While the standard DXA foot positioning does result in turning the femoral neck axis outward such that the femoral neck axis is more nearly orthogonal to the x-ray projection direction, the orthogonality of the femoral neck axis to the projection direction is not what is controlled with DXA. This orthogonality is, however, what is being controlled within CTXA.

Another cause of differences is projection geometry. CTXA uses a parallel beam projection geometry. While older DXA units also use a parallel projection geometry, most DXA units today use fan beam projection geometries that include a depth-dependent magnification attribute. Depending upon DXA unit design, there can also be a residual magnification component related to how far a patient's bone sits above, say, the DXA device table top. In such a situation, DXA BMD estimates can be influenced by patient positioning, including factors that may be difficult to control between DXA scans–such as significant weight loss/gain resulting in an inability to position a patient in the same manner between two scans. All of these are on top of algorithmic variations in core image processing steps used to define various anatomical landmarks and reference lengths used to standardize BMD measurement on a particular device. For example, thresholds of 15% of the maximum BMD across a femoral neck profile to define the lateral margins of the femoral neck (and so the femoral neck width) are likely to be very different in a DXA image, that includes a non-zero, noisy background that is a residual of soft-tissue background subtraction, in comparison to a CTXA image that includes essentially no soft-tissue background and essentially no soft-tissue background noise.

Well-established biases in BMD estimates from different devices are currently handled in clinical densitometry practice by reporting normalized BMD estimates and interpreting normalized proximal-femur BMD scores from all DXA units using the same guidelines. The use of T-scores is the prevalent normalization method in use today [14], although alternative methods for generating “standardized” BMD estimates have been proposed [21], [22]. When using the CTXA method, T-scores may be calculated by using conversion equations using the Hologic DXA-acquired NHANES III (National Health and Nutrition Examination Survey III) young normal data with conversion equations for the various anatomical regions [18]. This approach is similar to the adjustment made by DXA equipment manufacturers other than Hologic when using the NHANES III data to calculate T-scores [26]. Alternatively, young normal data may be gathered using the CTXA method in order to calculate T-scores. Although the normal subject data required for standardization of results by the computation of T-scores has been gathered for use with CTXA method and is in current clinical use that is not the subject of the study described here.

Osteoporosis represents a major public health issue, and there is a growing appreciation of the need for wider screening efforts. The recently revised and expanded recommendations by the U.S. Preventive Services Task Force (USPSTF) underscore the need for more screening [27]. The clinical utility of the CTXA hip method is in providing a BMD measurement where DXA may not be available due to space or financial constraints, but an underutilized CT scanner may be used such as in a rural healthcare facility. However, the disadvantage of a higher radiation dose must be considered in particular in younger individuals (e.g., peri-menopausal women).

The higher radiation exposure due to a dedicated QCT exam in comparison to a DXA study can be avoided by the re-use of a CT scan ordered for another reason. The use of QCT for BMD measurement at the lumber vertebrae and hip in conjunction with CT scans ordered for other reasons in the context of improving screening rates, has been explored in a number of studies [28]–[30] including for CT contrast-enhanced scans [31], [32]. For patients undergoing screening CT colonography (CTC), a potential opportunity exists for concurrent BMD screening without the need for any additional imaging, radiation exposure, or patient time [30]. Such dual-use of CT images could increase screening rates or, alternatively, preclude the need for DXA screening in some individuals. Because standard CT colonography exams include the pelvis, CTXA femoral neck measurement rather than lumber spine measurement can be made which provides access to the diagnostic classification using WHO T-scores thresholds and the use of FRAX for fracture risk calculation. The ability of the CTXA method to produce areal BMD and T-scores at the femoral neck which may be used as input to the WHO FRAX tool is particularly important as the field moves away from relying solely on BMD measurement for fracture risk prediction [33].

We have compared a method of deriving DXA-like 2D projections of integral bone from volumetric CT scans to DXA measurements. In principle, there is a large amount of useful 3D information discarded by this approach. In this study, we have presented results from the use CTXA Hip analysis to segment “cortical” and “trabecular” bone compartments which exploits the 3D nature of the acquired 3D image data. In terms of fully 3D analysis of the proximal femur, several methods have been developed [34]–[39]. Within each anatomic subregion, the density, mass, and volume are computed for the cortical and trabecular components as well as for the integral bone envelope. For trabecular BMD measurements, the precision of these kinds of methods *in vivo* was found to range from 0.6% to 1.1% depending on the volume of interest assessed [34]. Several of these approaches also facilitate geometric and structural analyses of the minimum femoral neck cross section, computing cross-sectional area, estimates of cortical volume and thickness, and moments of inertia for strength estimation. There has also been comparison made of hip structural analysis (HSA) parameters derived by DXA and QCT [40]. However, as has been stated before, the lack of normal data for standardization and the prevailing use of DXA T-scores for diagnostic categorization currently limit these volumetric 3D hip analysis methods to research and clinical trials application.

## Conclusion

We hypothesized that CTXA Hip BMD estimates could provide the same clinical utility as that afforded by DXA, and we believe our study results strongly support this hypothesis. High correlation of results from various combinations of DXA devices, along with consistency in measurement precision as characterized by SEE, interobserver variability, and assessment of long-term and short-term measurement precision have been accepted as a basis for using proximal femur results from all commercial DXA devices for clinical decision making within the context of any of numerous national and international clinical densitometry guidelines. Areal BMD and T-scores from CTXA Hip could provide standardized BMD measures from opportunistic osteoporosis screening using CT studies; or a method of osteoporosis screening in areas where DXA is unavailable. The 3D nature of QCT may also provide a more comprehensive estimate of bone strength parameters than similar analyses using DXA data.

## Supporting Information

Table S1 **Long Term In Vivo Precision CTXA vs DXA - Data.**
(XLSX)

Table S2 **Interobserver Comparison of CTXA BMD Estimates - Data.**
(XLSX)

Table S3 **Correlation of CTXA Hip and DXA BMD results - Data.**
(XLSX)
